# Ergothioneine: A Natural Antioxidant Claimed to Prevent Neurodegeneration

**DOI:** 10.3390/nu18213708

**Published:** 2026-11-08

**Authors:** Grzegorz Bartosz, Izabela Sadowska-Bartosz

**Affiliations:** Laboratory of Analytical Biochemistry, Institute of Food Technology and Nutrition, Faculty of Technology and Life Sciences, University of Rzeszow, 4 Aleksandra Zelwerowicza Street, 35-601 Rzeszow, Poland; gbartosz@ur.edu.pl

**Keywords:** ergothioneine, antioxidant, cognitive impairment, neurodegeneration, Alzheimer’s disease, Parkinson’s disease, mushrooms, signaling pathways

## Abstract

Ergothioneine (ET) is an exogenous thiol antioxidant present mainly in mushrooms and, in smaller amounts, in various other food products as well. Numerous beneficial health effects of ET have been reported, suggesting that ET is a vitamin-like compound. Evidence that ET prevents and inhibits the development of neurodegenerative diseases, especially Alzheimer’s disease and Parkinson’s disease, has been accumulated based on studies of cellular and animal models, as well as epidemiologic and interventional studies in humans. The mechanism of the neuroprotective action of ET remains to be elucidated. Apart from the direct antioxidant effect, ET was found to stimulate an endogenous antioxidant defense through the activation of Nrf2 signaling while also affecting other signaling pathways. The accumulating literature data suggest that an ET-rich diet could be recommended for the prevention of neurodegeneration in the general population, but such a recommendation, though noteworthy, is not yet adequately supported by the limited human intervention data, and further studies are needed.

## 1. Introduction

Although the mechanisms of human aging are still being explored, it is obvious that aging of the nervous system contributes significantly to the limitation of lifespans [1,2,3]. Neurodegenerative diseases contribute to the shortening of the lifespan and undoubtedly limit the healthspan as well [4,5]. Moreover, neurodegenerative diseases, compromising the independence and life quality of affected persons and their caregivers, are an important social and healthcare problem. Aging within societies aggravates the problem of neurodegenerative diseases, as most of which are age-related [6,7].

The term “neurodegenerative diseases” covers a range of disorders in which neurons are progressively lost in various regions of the central nervous system (CNS) [8]. This loss of neurons usually leads to the deterioration of cognitive, behavioral, or motor skills that occur in Alzheimer’s disease (AD), Parkinson’s disease (PD), Huntington’s disease (HD), multiple sclerosis, or amyotrophic lateral sclerosis (ALS) [9]. Mild cognitive impairment (MCI), a small decrease in memory and intellectual capacity that is worse than normal advancing with age and may precede, though not necessarily lead to, AD, is usually also included in this list [10]. Common to all neurodegenerative diseases is a set of hallmark pathological issues occurring in the brain, including neuroinflammation, oxidative stress, mitochondrial dysfunction, and impaired proteostasis, which collectively promote abnormal protein aggregation [8,11].

There is presently no cure for neurodegenerative diseases. Thus, preventing and slowing down their progress are the main ways to counteract their development. Oxidative stress accompanies neurodegenerative diseases and possibly contributes to their progression, so antioxidants have been proposed as a means to inhibit this process [12,13,14]. It would be desirable to prevent the onset of these diseases with compounds that can be constituents of everyday diet. Ergothioneine (ET) is a very promising compound in this respect.

Several reviews on ET’s effects on neurodegenerative diseases have been published recently [15,16,17,18,19]. The first review suggested that ET is a “stress vitamin”, focusing on its effects on vascular health, general anti-aging, and non-specific neuroprotection [15]. The second focused on the physiological distribution, transport, and fundamental presence of ET inside brain tissues [16]. The third review posed a highly optimistic hypothesis that neurodegenerative diseases are caused by a dietary lack of ET and strongly advocated for ET-rich diets [17]. The next review provided a classic systematic review, mapping and cataloging existing data regarding ET’s effects on cognition and neurodegenerative diseases [18]. The last review offered a highly specific, mechanistic look at ET as a multifactorial protectant but limited its scope almost entirely to PD [19]. Our critical narrative review presents the properties of ET that are relevant to neuroprotection; the proposed mechanisms of ET’s action that may or may not be related to its antioxidant action; and critically evaluates the evidence for the beneficial effects of ET in cellular and animal models, mainly of AD and PD, and its neuroprotective effects reported by epidemiological data and human intervention studies, including newly published data. We also discuss dietary sources of ET, its blood levels, and the effects of supplementation.

## 2. Properties of Ergothioneine

Ergothioneine, or {5-[(2S)-2-carboxy-2-(trimethylazaniumyl)ethyl]-1H-imidazol-2-yl}sulfanide, is a thiol/thione derivative of histidine, present primarily in the thione form at neutral pH. ET is a potent water-soluble antioxidant.

Various standard redox potential values have been reported for ET; a recent study proposed a value of 0.45 ± 0.02 V and the following sequence of reducing strengths at physiological pH: glutathione ≈ cysteine ≫ ascorbate > α-tocopherol ≈ ergothioneine > tyrosine [20]. ET is thus a relatively weak reductant; nevertheless, it can scavenge biologically important reactive oxygen species (ROS), including hydroxyl radical (•OH), ozone (O_3_), peroxynitrite (ONOO^−^), singlet oxygen (^1^O_2_), hypochlorous acid (HOCl), and hypobromous acid (HOBr), and reduce higher oxidation states of heme proteins [21]. ET quenches singlet oxygen at higher rates compared to other thiols [22]. It has been suggested that ET’s main function is the prevention of singlet-oxygen-induced damage [23].

**Figure 1:**
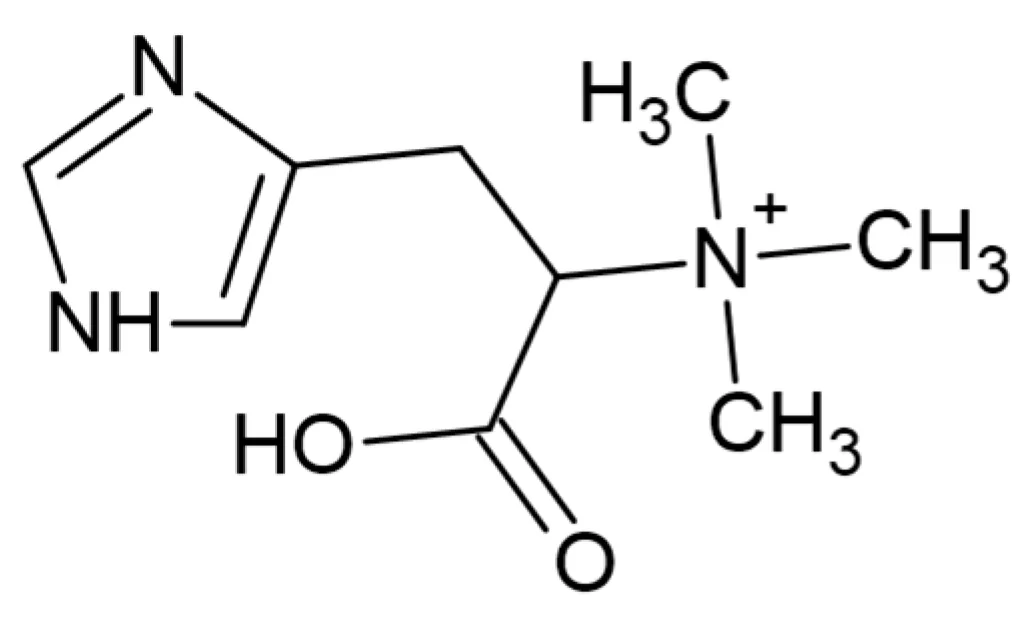
Structure of hercynine (3-(1H-imidazol-5-yl)-2-(trimethylammonio)propanoate), a metabolic precursor and metabolite of ergothioneine.

The first product of ET oxidation is ET disulfide, or a mixed disulfide with another thiol [24]. Further oxidation products can also be formed [25]. Regeneration of some oxidized forms of ET (ET disulfide and 5-oxo-ET) can be rapidly accomplished non-enzymatically using ascorbic acid [26] or enzymatically with either glutathione reductase (but only in the presence of glutathione) or thioredoxin reductase [27]. ET’s antioxidant action has an interesting peculiarity. ET disulfide is unstable at physiological pH and undergoes disproportionation. In this reaction, 3 mol of ET and 1 mol of hercynine (Figure 1) are formed from 2 mol of the disulfide [25]. Such partial ET regeneration from the disulfide does not require participation of reducing agents (a reaction that can be compared to the feat of Baron Münchhausen pulling himself out of a swamp by his own hair, though only by ^3^/_4_ and not as a whole).

ET is a good chelator of divalent metal ions, such as Cu^2+^, Hg^2+^, Zn^2+^, Cd^2+^, Co^2+^, Ni^2+^, and Fe^2+^, and forms complexes at a ratio of 2 mol ET/mol with a divalent metal ion [28,29]. Chelation of iron and copper ions to form redox-inactive complexes prevents their participation in the Fenton reaction, generating hydroxyl radicals [30] and preventing copper-dependent oxidative damage to biological macromolecules [31].

ET, being less prone to reduction, is more stable in vivo than glutathione (GSH), and it is more resistant to autoxidation and heating [32,33,34,35]. ET does not degrade during prolonged storage, which facilitates its application. The use of ET as a preservative for food products has even been proposed. An additional advantage of ET in this respect is preventing the development of melanosis, owing to the metal-chelating properties of this compound [36].

## 3. Dietary Sources of Ergothioneine

The biosynthesis of ET has been believed to be unique to fungi, including many yeasts and a few bacteria [37,38], and it has not been observed in higher plants or animals [39]. Fungi synthesize ET from histidine and cysteine with the help of two enzymes that are products of the *EGT1* and *EGT2* genes. The *EGT1* enzyme performs two functions, catalyzing the triple methylation of histidine to hercynine and then conjugating hercynine with cysteine and oxygen to form hercynylcysteine sulfoxide. Subsequently, *EGT2* cleaves the C-S bond, forming ET [40]. Bacteria have two separate single-domain genes coding these two enzymes that together catalyze the same reactions as *EGT1* [41]. Plants were believed to obtain ET exclusively via the uptake of exogenous ET, synthesized by fungi and/or bacteria. It has been suspected that innovative agricultural practices that affect the soil mycobiome and bacteriome can diminish ET levels in edible plants, leading to decreased dietary ET uptake. Plants grown hydroponically may have low or null levels of ET [42,43,44]. However, a recent report showed the presence of *EGT1* and *EGT2* genes in streptophyte algae, bryophytes, lycophytes, monilophytes, and gymnosperms, but the *EGT1* gene was absent in angiosperms, and thus they are unable to synthesize ET. Yet angiosperms can apparently obtain ET so easily from fungi and bacteria in the soil that the loss of the *EGT1* gene did not have any negative consequences for them. In the alga *Klebsormidium nitens* and the moss *Physcomitrium patens*, the ET content increased in response to high light and heat stress [41].

Animals consume ET, which seems to be a vitamin-like compound for them [15], in their diets. Mushrooms, which are able to synthesize ET and thus have a higher content of this compound, are the main dietary source of ET [45,46,47]. About 95% of total ET intake in the typical European diet is due to mushroom consumption [48]. Amongst edible mushrooms, the porcini mushroom (*Boletus edulis*) [35], lion’s mane (*Hericium erinaceus*) and oyster mushrooms (*Pleurotus ostreatus*) were found to be richest in ET, with cordyceps (*Cordyceps militaris*) and shimeji (*Hypsizygus tessulatus*) being the next ET-rich species [49,50,51]. However, the ET content of different batches varies greatly depending on the growth conditions of the fungi. In cultivated mushrooms, ET content depends on cultivation practices, including the substrates [52], soil conditions, and cultivation methods used [53]. Detectable levels of ET are also present in fruits and vegetables (e.g., garlic and asparagus); nuts, beans, corn, and spices (e.g., basil, ginkgo, and cumin); *Spirulina*; and also in milk, fish, eggs, and fermented soy products, such as tempeh [50]. The eggs of hens whose feed was supplemented with the ET-producing fungus *Neurospora crassa* were demonstrated to have an elevated ET content [54].

The data on ET uptake are limited. Americans were found to consume less ET (ca 1.1 mg/day) than the inhabitants of four European countries (Finland, 1.3; France, 2.2; Ireland, 3.6; and Italy, 4.6 mg/day) [55,56]. Mean dietary ET intake in China was 0.043 mg/kg body weight per day, although big differences between various regions were noted [57]. Interestingly, very high blood ET levels were found in the Inuit, who apparently derived ET from meat and marine foods, including Beluga whales [58,59].

## 4. Bioavailability of Ergothioneine

High bioavailability of ET has been found across plants, *Drosophila*, rodents, and humans. All these organisms retain ET avidly [60]. Feeding *Drosophila* with 1 mM ET in the diet for 25 days increased their ET concentrations in the brain and body 10-fold and 40-fold, respectively [60]. In mice administered ET orally (35 and 70 mg/kg/day), ET levels increased in all tissues by day 7 and increased further until day 28, when the brain ET content grew by approximately 2.9-fold [61].

Human studies confirmed the high uptake and retention of ET administered orally to healthy volunteers. ET is absorbed from the gastrointestinal tract, distributed by the blood, and then accumulated in tissues. ET is reabsorbed in the kidneys, so its excretion in urine is very low, but some ET is excreted in feces [19,35,61,62,63].

The concentrations of ET in the blood plasma and other body fluids are much lower than those in erythrocytes and, thus, in whole blood and tissues [15,30,34,64]. The blood levels of ET found in various studies, as well as the effects of supplementation, are shown in Table 1. Exceptionally high whole blood ET concentrations were found in the Inuit population of Nunavik (Quebec). ET concentrations were higher in the blood of females than males and increased progressively with age, from the age group of 16–19 y up to the group of 60+ y. The dietary questionnaire pointed to the association of ET levels with the rate of consumption of Arctic charr (*Salvelinus alpinus*), other fish, dried beluga meat, caribou meat, and hen eggs [59].

ET concentrations in the whole blood, plasma, and urine of subjects who received a daily ET dose of 5 mg/day for 7 days were ca 12.5 µM (the value of 125 µM reported in the text is obviously erroneous), 2 µM, and 20 nmol/mmol creatinine higher than those at baseline, respectively, indicative of a high ET uptake. Less than 4% of administered ET was excreted in urine, and whole blood ET levels rose over the entire study duration up to 28 days (and possibly further), indicating the high retention of ET in humans [63]. In another clinical trial involving elderly subjects with MCI, prolonged consumption of ET over a year resulted in a continuous increase in blood plasma ET levels up to ca 7 µM after 56 weeks, with no sign of saturation [73].

ET can cross the blood–brain barrier (BBB) and accumulate in the brain [17,19,35,76]. ET was detected in the brain of various mammals. It was found to be present in the cerebellum in amounts ranging from about 3.0 µg/g in cats to 10 µg/g in guinea pigs. Its amounts in the cerebral hemispheres were much smaller, ranging from <0.1 µg/g in cats to 1.6 µg/g in guinea pigs [77,78,79,80]. Large amounts of ergothioneine (about 30 µg/g) were found in the optic nerves of rabbits [79]. Interestingly, compared to its basal level, the brain also exhibited markedly higher amounts of hercynine, an oxidative metabolite of ET [25], following ET supplementation [61], indicating that ET is oxidized in the brain as a result of its antioxidant activity.

A tacit assumption is that the circulating ET concentrations reflect its accumulation and biological activity in specific brain regions, which may not exactly be the case. It was found experimentally that ET supplementation of mice led to a 3.3-fold increase in its concentration in whole blood, resulting in 4.8-fold, 3.0-fold, 2.9-fold, 2.6-fold, 2.5-fold, 2.5-fold, 2.3-fold, 1.9-fold, and 1.5-fold increases in the kidney, large intestine, eye, lung, liver, heart, small intestine, and spleen, respectively. The kinetics of ET accumulation varied in different organs; e.g., in the liver and lungs, maximum levels were reached after 7 days of a 28-day experiment [61]. A PET study of short-time ^11^C-ET accumulation (up to 30 min) after intravenous injection showed the highest accumulation in the kidneys and liver. In the brain, the accumulation was higher in the cerebellum and cortex than in the hippocampus, thalamus, and striatum [81].

Detailed knowledge of metabolic changes necessary for the sleep state in the brain is missing. ET levels were significantly lower in the cerebellum during sleep and were higher in the plasma of the wake-state mice compared to sleep states [82].

ET passes from mother to child through the placenta and is present in breast milk [21,63,83].

The gut microbiome may play a role in regulating the uptake of ET. Some intestinal bacteria were reported to contain ET (although ET biosynthesis in these species was not directly demonstrated) [84,85] and potentially may contribute to the regulation of host ET levels. *Lactobacillus reuteri*, a gut commensal bacterium widely promoted as a probiotic to promote human gut health that typically colonizes the small intestine, was claimed to produce ET [84]. However, it was demonstrated that *L. reuteri* does not produce ET but avidly accumulates it [86], which points to a more complex regulation of ET levels by gut microbiota. *Helicobacter pylori* cannot synthesize ET but takes it up avidly; apparently, ET helps to protect this pathogen against ROS generated by human phagocytes [87,88]. It was suggested that some components of the microbiome (Cyanobacteria and related species) in mice could produce ET [85], but these bacteria are not abundant in the human gut [89]. However, the human gut contains not only a bacteriome but also a mycobiome, a set of fungi accounting for no more than 0.1% of microorganisms detected in the human gut; nevertheless, some of them are perhaps able to synthesize ET, interact with bacteria, and be a minor ET source for the host [90].

## 5. Transport of Ergothioneine

ET from the diet is absorbed through the intestine. Its distribution in the body is governed by the organic cation transporter novel 1 (OCTN1), a product of the *SLC22A4* gene [91]. OCTN1 is a member of the solute carrier 22 (SLC22) family. These proteins transport diverse cationic and zwitterionic compounds. Several endogenous organic cations, such as acetylcholine, γ-butyrobetaine, carnitine, and carnitine derivatives, as well as some gut microbiota by-products, were identified as the transport substrates of OCTN1 [92,93]. However, ET is likely to be the major physiological substrate of OCTN1. OCTN1 also transports selenenonine, a selenium analog of ET [93]. OCTN1 is highly expressed in most, if not all, tissues and vital organs in mammals. Appreciable expression levels were found in the brain, lung, skin, appendix, tonsils, and bone marrow [94]. Apparently, this transporter ensures the specific uptake and accumulation of ET in various tissues, including the brain [19].

ET transport by OCTN1 represents a secondary active transport dependent on Na^+^, utilizing the transmembrane Na^+^ gradient (higher Na^+^ concentrations outside than inside cells) and thus, the tendency of Na^+^ to enter the cytoplasm. Coupling the Na^+^ influx to ET transport allows for ET accumulation inside the cells, even if its concentration there is higher than outside the cells. OCTN1 activity enables avid ET uptake in the intestines; high ET retention in the proximal renal tubules, thereby facilitating renal reabsorption; and the presence of ET in most tissues. After administration of ET to *SLC22A4*-deficient mice, this compound was detected in the blood plasma, but its levels in the tissues were below the limits of detection [95].

As mentioned, the level of ET in erythrocytes is much higher than in plasma. Still, the presence of OCTN1 was not detected in mature erythrocytes and reticulocytes, suggesting that ET is accumulated in erythropoietic precursors of erythrocytes and remains there during their differentiation into mature red blood cells [96].

In the brain, the highest expression of OCTN1 was found in the medulla oblongata, midbrain, and spinal cord [97].

One of the obstacles to the effective pharmacologic treatment of central nervous system diseases is the BBB. Generally, BBB is poorly permeable to hydrophilic compounds and easily permeable to hydrophobic ones. ET shows high BBB permeability despite being hydrophilic. Interestingly, the presence of OCTN1 in the human BBB has been suggested but not unequivocally proven [21,97]. The functional expression of OCTN1 has been detected in neurons [98], neural stem cells [99], microglia [100], and cerebral vascular endothelial cells [101]. In the cerebral cortex, the level of OCTN1 is much higher in astrocytes than in neurons, suggesting its importance for astrocytes [19,94]. Within cells, OCTN1 is functionally expressed in the plasma membrane. Its presence in the mitochondria has been both reported [102,103] and denied [104], but mitochondria isolated from OCTN1 knockout mice tissues also accumulate ET [104].

Genetic polymorphisms of the *SLC22A4* gene significantly affect OCTN1 expression, substrate affinity, and thus, the pharmacokinetics of ET [105,106]. The proportion of polymorphic variants varies with ethnicity [107,108] and may contribute to the differences in blood ET levels found in various populations (Table 1). Moreover, epigenetic modifications, hormones, and cytokines modify OCTN1 activity, so it may be altered in response to immune modulation and disease progression [93].

Circadian changes in the intestinal expression of OCTN1 were found in mice. Such changes can affect ET uptake from the diet [109]. Administration of 1α,25-dihydroxyvitamin D_3_ was found to alter OCTN1 levels in various rat tissues [110]. The calcium channel blocker verapamil inhibits OCTN1 [111].

Surprisingly, the genetic deficiency of OCTN1 does not evoke significant phenotypes and did not cause functional abnormalities in the body [112]. This finding can be explained by the existence of alternative means of ET transport. An alternative explanation would suggest that ET is, in fact, not indispensable physiologically.

Another human transporter, SLC22A15, does not play a role in the intestinal absorption of ET, but its level is high in the brain. Creatine seems to be the main substrate of this transporter. ET may also be a substrate, but its affinity for SLC22A15 is low (K_m_ of ca 400 μM vs. ca 21 μM for OCTN1), and V_max_ is also lower compared to OCTN1 [113,114,115].

## 6. Ergothioneine in Neurodegenerative Diseases

Several observational studies have pointed to an inverse association between blood ET levels and the hazard or presence of certain neurodegenerative diseases.

Whole blood and blood plasma levels of ET in elderly persons (60 y+) were found to decrease significantly [67]. An earlier study pointed to a tendency for an increase in the ET content in erythrocytes of males over 51 y of age [65], but the results were obtained with a colorimetric method [116], which is less reliable than modern methods based on HPLC separation. Moreover, the age range of the “51+” group was not specified [67], so these two sets of data are not necessarily mutually exclusive.

Subjects with MCI had significantly lower total blood ET than age-matched subjects (150.9 ± 18.6 vs. 232.5 ± 27.5 ng ET per mg hemoglobin) [67]. Another study found lower ET levels in people with dementia (0.588 µM) and with cognitive impairment without dementia (0.798 µM) when compared to those with no cognitive impairment (1.197 µM) of a similar age (about 70 y). Low plasma ET levels correlated with decreased global cortical thickness and hippocampal volume, white matter hyperintensity and cortical infarcts. It was suggested that a low level of plasma ET might be an early biomarker for cognitive impairment and dementia. No differences in the blood plasma ET concentrations were found between patients with AD and vascular dementia, but both groups had lower ET concentrations in comparison with controls with no cognitive impairment [117]. Lower serum ET concentrations were found to correlate with increasing psychological distress in older adults [68].

PD patients were found to have serum ET levels 45% lower than healthy controls [118]. A later study confirmed significantly lower mean plasma ET levels in PD patients and lower plasma ET levels in individuals with other neurodegenerative or age-related disorders compared to normal age-matched controls [17].

An analysis of the relations between the levels of 991 blood metabolites and cognition showed the highest association for ET, with worse cognition being associated with lower ET levels [119]. Other metabolomic studies revealed an association of plasma ET levels with the global cognitive score [120], and decreased ET levels in the whole blood of patients with dementia [121]. Frailty is a state of age-related mental and physical functional decline, in which both cognitive functions and physical performance are diminished. Blood ET levels were lower in patients with frailty and cognitive dysfunction, while sarcopenia and restricted mobility did not decrease blood ET levels [122,123]. It was suggested that declining ET levels may be a risk factor for neurodegeneration [67] and an early potential peripheral biomarker for cognitive impairment [112]. In the Boston Puerto Rican Health Study (314 participants), blood plasma ET levels were associated with higher global cognitive scores [124].

However, correlation data do not provide the ground for conclusions about a cause-and-effect relationship, and it is difficult to judge whether lowered ET levels are a cause or consequence of neurodegenerative diseases [64]. Increased oxidative stress in these diseases may lead to an enhanced consumption of ET as an antioxidant.

Longitudinal studies apparently speak in favor of the role of ET deficiency in the development of cognitive impairment. A follow-up longitudinal study on 470 individuals showed that lower ET levels in individuals without cognitive impairment were associated with a faster decrease in global cognitive function and in language, attention, memory, and locomotor functions [125]. Similar conclusions were reached in a prospective longitudinal analysis of 1344 Japanese community residents aged 65 y+ without dementia at baseline who were followed for 11.2 y on average. In this cohort, higher serum ET concentrations were associated with a lower risk of developing all-cause dementia, AD, and non-AD dementia [70].

The multi-omics analysis of the Alzheimer’s Disease Neuroimaging Initiative cohort revealed that lower blood ET levels in individuals with MCI were associated with a 12% higher rate of AD progression within two years [126].

Sleep and circadian disruption are common in AD, PD, and Huntington’s disease [127,128]. ET may mitigate age-related decline in circadian rhythmicity, provided that the results from an in vitro study of mouse embryonic fibroblasts [129] can be extrapolated to the whole organism.

The formation of hercynine from ET is a measure of the oxidative metabolic utilization of ET. A significant increase in the L-hercynine/ET ratio was found in dementia [117]. Higher L-hercynine/ET ratios were found to mitigate the positive association between plasma phosphorylated tau protein (*p*-tau) levels and cognitive decline. Individuals with a high amyloid burden had a higher risk of cognitive decline in comparison with individuals of lower amyloid burden when the hercynine/ET ratio was low. Thus, the blood plasma hercynine/ET ratio has also been proposed as a novel biomarker of cognitive resilience [130].

However, there are also data not fitting the idea of the inverse relationship between ET uptake or its level and the risk of neurodegeneration. One is the lack of differences in plasma ET concentrations between patients with AD and vascular dementia mentioned above [117]. Moreover, blood plasma ET concentrations in elderly (65–90 y) persons without dementia or other pathological cognitive impairment that had low neocortical amyloid loads (Aβ−) and high neocortical amyloid loads (Aβ+) were reported not to differ [74]. Surprisingly, an increased serum ET concentration was found to be a risk factor for AD [131]. The authors of this report attempted to reconcile these results with other data by proposing that the high levels of ET in the serum of AD patients might be a result of diminished uptake by the brain. New data are needed to shed more light on this question.

Since mushrooms constitute the main dietary source of ET in most of the world, the relation between mushroom consumption and cognitive performance, as well as with general health, has been analyzed.

An apparent negative association has been noted between the estimated ET consumption in the US and four European countries and the mortality rates due to AD and PD, and a positive correlation was found between ET intake and life expectancy [56]. However, this correlation was based on five pairs of data only. A cohort study of 13,230 Japanese individuals, aged ≥65 y, showed that a higher mushroom consumption was associated with a decreased risk of incident dementia [132]. Data from the 2011–2014 National Health and Nutrition Examination Survey showed that a higher mushroom intake among older adults in the USA was positively associated with cognitive performance [133]. A study of mushroom intake in 663 elderly individuals from the Diet and Healthy Aging study in Singapore found an association between the extent of mushroom consumption and a decreased risk of MCI [134]. A study performed in China, including 2203 middle-aged and older adults (≥55 y), revealed a positive association between mushroom consumption and global cognition, as evaluated by the Digit Symbol Substitution Test and Trail Making Test—B. An increase in mushroom consumption by 10 g per day was associated with 12% lower odds of MCI [135]. Among 248 Chinese older adults, a high mushroom consumption was associated with a low risk of MCI, which was estimated with the Montreal Cognitive Assessment (MoCA) score. This effect was associated with, and probably partly dependent on, the lowering of inflammatory mediators, including IL-6 and hs-CRP [136].

Nevertheless, it should be taken into account that dietary surveys are not accurate, as ET contents differ in various mushrooms, and, in addition to ET, mushrooms contain a plethora of other compounds that might affect cognition [48,137,138].

The gut–brain axis in AD and PD has aroused interest. It has been discussed that the number and ratios of intestinal bacteria that contain ET may decrease during the onset of neurodegenerative diseases, which may diminish the absorption of ET and its blood levels [139,140,141,142].

## 7. Effects of Ergothioneine Administration

The association between lower concentrations of circulating ET and elevated risks of neurodegenerative diseases allows us to expect that the restoration of proper ET levels may be helpful in the prevention and treatment of these diseases, such as AD or PD. This idea has been tested in cellular and animal experiments and, finally, in human interventional studies.

### 7.1. Cellular Experiments

ET was demonstrated to exert protective effects in various cellular models of neurodegenerative diseases, mainly due to its antioxidant activity.

SH-SY5Y neuroblastoma cells differentiated with retinoic acid and acquiring neuron-like characteristics are used as an in vitro AD model under conditions of Aβ exposure to amyloid β peptides (Aβ) and as a model of PD when treated with α-synuclein, 6-hydroxydopamine (6-OHDA) or 1-methyl-4-phenylpyridinium (MPP^+^). ET significantly suppressed the Aβ25–35 oligomer-induced cytotoxicity and the levels of *p*-tau in differentiated SH-SY5Y cells and cultured hippocampal neurons [143]. In SH-SY5Y cells treated with α-synuclein, ET decreased the levels of ROS and malondialdehyde (MDA) and augmented the levels of GSH and superoxide dismutase (SOD) [144]. ET protected dopaminergic neurons derived from induced pluripotent stem cells (iPSCs) and SH-SY5Y cells against the 6-OHDA-induced loss of viability, a decrease in mitochondrial potential, ATP loss, augmentation of ROS levels, a decrease in tyrosine hydroxylase activity, and elevated protein carbonylation [145].

ET dose-dependently protected immortalized mouse hypothalamic neurons (GT1-7 cells) against 6-OHDA- and MPP^+^-dependent death by suppressing ROS overproduction. The protection by ET was attenuated by verapamil, an inhibitor of OCTN1 [146].

In human brain microvascular endothelial cells, ET decreased ROS levels and increased the viability of cells challenged with high glucose and oxidants [101]. ET protected hCMEC/D3 human BBB endothelial cells against 7-ketocholesterol-induced injury (with increased levels of intracellular free calcium, cellular, and mitochondrial ROS and a decrease in mitochondrial membrane potential and ATP levels) [147]. ET augmented the expression of synapsin I, a marker of synapse formation, in cultured hippocampal neurons [148].

Oxaliplatin is used to treat colorectal cancer and other malignancies; however, its clinical use is often limited by oxaliplatin-induced peripheral neuropathy. ET attenuated the oxaliplatin-induced increase in ROS and MDA levels and inhibited cytotoxicity and apoptosis in differentiated SH-SY5Y cells, all without affecting the oxaliplatin cytotoxicity to HCT-116 and DLD-1 colorectal cancer cell lines [149].

It should be noted, however, that ET concentrations used in most of these cellular studies were pretty high, reaching or exceeding 1 mM, exceeding those attainable in vivo, and the protective effects observed could be due to a non-specific antioxidant action that probably could be achieved using other antioxidants.

### 7.2. Animal Experiments

The neuroprotective properties of ET have been demonstrated in various pre-clinical models of PD and AD, including Aβ-overexpressing *C. elegans* as a model of AD [150]; α-synuclein-expressing transgenic *C. elegans* as a model of PD [144]; parkin-null and LRRK2 G2019S transgenic *Drosophila* as PD models [60]; α-synuclein-expressing mice as a model of multiple system atrophy [151]; 6-OHDA- [60] and 1-methyl-4-phenyl-1,2,3,6-tetrahydropyridine (MPTP)-induced [144,152] PD models in mice; an Aβ-induced mouse model of AD [153]; 5κκ × FAD mice (genetically modified to have five human genes that cause AD) [154] and APP/PS1 mice, expressing mutated human amyloid precursor protein (Mo/HuAPP695swe) and mutant presenilin 1, as models of AD in mice [155,156].

ET treatment has been shown to augment neuronal survival and ameliorate cognitive deficits in various animal models of AD and PD. Overexpression of human Aβ in transgenic *C. elegans* leads to paralysis and premature death. This Aβ toxicity was ameliorated by ET in a dose-dependent manner in this AD model: ET decreased Aβ oligomerization and oxidative stress and extended the mean and maximum lifespan of the Aβ-overexpressing nematode by 6% and 11%, respectively, at the concentration of 5 mM [150]. In *C. elegans* overexpressing α-synuclein (a PD model), ET reduced the accumulation of α-syn plaques, protected dopaminergic neurons, improved behavioral functions, reduced oxidative stress, and prolonged the lifespan by up to 50% [144].

In *Drosophila* models of PD, ET treatment ameliorated the pathological phenotypes of parkin and LRRK2 PD mutant flies. ET effects included improved locomotor function and the preservation of their dopaminergic neuronal number and mitochondrial integrity [60].

Most studies were performed on rodent models. ET was found to ameliorate cognitive and behavioral deficits in various transgenic or toxin-induced models of AD and PD in mice.

β-Amyloid peptides (Aβ) cause neurotoxicity and contribute to the development of AD. ET given orally (0.5 or 2 mg/kg body weight) for 16 days to mice before treatment (i.c.v. single dose of Aβ1–40 in the hippocampus) and for 39 d after treatment prevented Aβ accumulation in the hippocampus and brain lipid peroxidation while maintaining the glutathione/glutathione disulfide (GSH/GSSG) ratio and SOD activity in the brain. Moreover, ET ameliorated the loss of memory and learning abilities in mice, decreasing the escape latency and augmenting the frequency of successful avoidance in the water maze and long-memory avoidance tests, respectively [157].

Similarly, treatment of APP/PS1 transgenic mice with ET (100 mg/kg bw, every day for 12 weeks) resulted in an increase in alternation rate in the Y-maze test and a reduction in escape latency in the Morris water maze test. ET decreased the levels of pro-apoptotic proteins like cleaved caspase 3 and Bax, and enhanced the levels of anti-apoptotic Bcl-2. The joint administration of ET and lactoferrin improved cognitive function and alleviated typical AD pathological characteristics, with decreased Aβ levels in the hippocampus, pyramidal cortex, and thalamus; decreased levels of Aβ1-40 and Aβ1-42 in the plasma and cortex; and reduced Aβ aggregation and tau protein phosphorylation [155].

ET treatment (25–50 mg/kg) of young (2-month-old) 5XFAD mice for 8 weeks by oral gavage prevented a cognitive deficit and improved associative memory, as evaluated by enhanced freezing behavior in the fear condition test. The treatment decreased amyloid plaques, ameliorated oxidative stress, and improved glucose metabolism [158].

In a related study employing APP/PS1 mice, ET treatment (40 mg/kg) administered in the drinking water for 6 months significantly improved cognitive function, limited neuronal damage, and reduced Aβ accumulation and microglial overactivation and TNF-α expression in the mouse brain. Increased spontaneous alternation in the Y-maze test and increased novel object exploration were observed. These results suggest the potential of ET to improve memory function and alleviate AD symptoms [156]. Moreover, ET decreased escape latency (by 49–85%) and distance traveled in the reference memory task (by 53–69%) in an Aβ-induced C57BL/6J mouse AD model [153].

In a 6-OHDA mouse model of PD, ET (8 mg/kg·day) administered for 4 weeks resulted in significant attenuation of motor impairments, with a reduction in dopaminergic neuronal loss in the substantia nigra pars compacta, an increase in the firing rates of neurons in the primary motor cortex (M1), reduced pathological β oscillations (13–30 Hz) and β-γ phase-amplitude coupling in both the M1 and subthalamic nucleus, an increase in the spine density of pyramidal neurons, and elevated levels of glutamate receptors in the M1 [159].

In a 6-OHDA unilateral lesion mouse model, ET treatment diminished the loss of dopaminergic neurons in the substantia nigra by about 50%. This protection of dopaminergic neurons resulted in improved behavioral indices, with reduced rotational behavior from seven turns to just two turns per minute and increased rotarod latency by 100 s [60]. These protective effects were attenuated by the inhibition of OCTN1 with verapamil [60,145,146], confirming that ET acts intracellularly.

Similar neuroprotective effects were found in the MPTP mouse model of PD, where ET treatment increased the fraction of surviving dopaminergic neurons after MPTP administration from 48% up to 74% [144]. In the same model, ET restored dopamine levels, decreased ROS, alleviated the MPTP-induced dysfunction of locomotor activity and exploratory drive, as measured by the open field test, and of motor coordination function by the rotarod test in mice. The protective effects were comparable to those of Rasagiline, a clinically approved drug for PD [152].

A 6-week administration of ET-rich king oyster mushrooms *Pleurotus eryngii* ameliorated brain atrophy in Aβ-induced C57BL/6J mice; diminished escape latency and distance traveled in the reference memory behavior test; reduced memory deficits in the probe and T-maze tests; and diminished Aβ plaque deposition, the amount of brain *p*-tau, and the levels of MDA and protein carbonyls in the brain [153].

Administering extract from a high ET-producing strain of the lion’s mane mushroom *Hericium erinaceus* (5.76 mg/kg for 90 d) or pure ET (1.44 mg/kg) to mice reduced Aβ aggregation and tau phosphorylation; improved cognitive function, spatial learning, and memory; lowered oxidative stress markers; and increased antioxidant indicators (especially GSH levels and catalase activity) in the brain of transgenic APP/PS1 mice [160].

D-Galactose treatment impaired learning and memory abilities in mice. ET (0.5 mg/kg body weight given orally for 88 days) reduced β-amyloid accumulation in the hippocampus, lowered lipid peroxidation, maintained the GSH/GSSG ratio and SOD activity in the brains of D-galactose-treated mice and, especially in combination with melatonin, protected against learning and memory deficits, probably mainly through the attenuation of oxidative damage [161].

Multiple system atrophy is characterized by the accumulation of abnormal α-Syn species, among them being toxic α-Syn oligomers, in the central nervous system. In a mouse model of multiple system atrophy, intranasally administered ET (10 µg once per week for 4 weeks) enhanced short-term spatial memory linked to hippocampal activity, reduced α-Syn 17 phosphorylation in the cerebral cortex, increased the expression levels of α-Syn monomer, reduced α-Syn dimer levels, and decreased the level of aggregated α-Syn in the hippocampus [151].

Spinal muscular atrophy (SMA) is a genetic disorder characterized by the loss of spinal motor neurons. ET administered in the drinking water (20 µg/d) to pregnant and lactating mice bearing SMND7 embryos increased the survival and locomotor abilities of pups on the 11th day. ET also stimulated mitophagy in isolated diaphragm muscle [162].

Presbycusis (age-related hearing loss) is a communication deficit and neurodegenerative disorder common in the elderly. This CBA/CaJ mouse strain has become the gold standard in research on age-related hearing loss (ARHL). Mice of this strain retain good hearing throughout most of adulthood, and their slow, high-frequency hearing loss in old age closely mirrors the natural aging process of the human hearing system. ET administered to 25–26-month-old CBA/CaJ mice for 6 months (35 or 70 mg/kg bw, once per week, ip) attenuated presbycusis in male but not female mice; this difference was due to a higher accumulation of ET in males [163].

Rat social defeat stress is used as a model for major depressive disorder. Administration of ET (ca 30 mg/kg per day) for 3 weeks prevented social defeat stress-induced depressive behaviors [84].

A side effect of the anti-tumor drug cisplatin is its neurotoxicity. Memory and learning abilities were impaired in mice administered intraperitoneally with cisplatin. Administration of ET (2 or 8 mg/kg bw for 56 d, orally) to cisplatin-treated mice significantly attenuated the decline in their memory and learning abilities [164].

Administration of ET (20 mg/100 g diet) of golden oyster mushroom (*Pleurotus citrinopileatus*) extract containing 1.2% ET (10% of the feed) for 2 weeks to 5–8-week-old male mice reduced the effects of immobilization stress, exerted an anti-anxiety effect and an antidepressant-like effect, decreased the immobility time in the forced swimming test and in the tail suspension tests, and promoted neuronal differentiation [165].

ET administration to healthy mice protected against age-related worsening of central nervous system functioning as well, suggesting that ET can improve cognitive function, both in cognitive impairment and under normal conditions [112].

Mice administered ET (1–50 mg/kg, orally) three times per week for two weeks showed a longer exploration time for a novel object than for a known one, and for a spatially moved object than an unmoved object with a significantly higher discrimination index [148].

Orally ingested ET was found to promote neurogenesis in the hippocampal dentate gyrus from neural stem cells and exert antidepressant-like effects in mice [166].

Maintaining young mice on an ET-free diet from 3 to 8 weeks of age resulted in impaired learning and memory abilities. Subsequent oral administration of ET (2 and 20 mg/kg) for two weeks (on days 0, 2, 4, 7, 9, and 11) resulted in a recovery of the hippocampal ET levels in ET-deficient mice to concentrations similar to those of control mice fed a normal diet, and it reversed the impairment in object recognition and spatial memory caused by ET deficiency [167].

ET administered orally (1–50 mg/kg) three times per week for two weeks augmented the number of mature spines in the hippocampus, improved memory and learning ability in healthy mice, and amended their cognitive function, as assessed by the object recognition test [148,168].

In summary, data accumulated over the last few years has provided evidence of neuroprotective effects of ET supplementation in various in vitro and in vivo models of PD and AD, as well as in other cases of neurodegeneration. Though these results seem quite convincing, the studies concerned model systems and often employed very high doses of ET, far exceeding the reported consumption in human populations.

### 7.3. Human Interventional Studies

Administration of ET to human volunteers (5 or 25 mg/day for up to 35 days) revealed decreasing trends (though no statistically significant differences) in blood plasma biomarkers of oxidative damage and inflammation, such as protein carbonyls, C-reactive protein, and urinary 8-hydroxy-2′-deoxyguanosine. No adverse effects of ET administration were observed [63].

Oral administration of ET (8 mg/day) for 16 weeks to healthy subjects aged 40–64 years, with an awareness of memory or attention decline, resulted in a significant improvement in subjective sleep quality [69]. Volunteers with anxiety and sleep complaints given 20 mg ET/day for 4 weeks considerably ameliorated their sleeping difficulties [71]. As sleep disturbances are often observed several years before the occurrence of motor symptoms, this finding suggests (but by no means proves) that ET supplementation initiated at early stages of sleep difficulties may not only ameliorate these difficulties but also prevent or delay the onset of PD [19]. A 16-week administration of ET (10 or 25 mg daily) to healthy subjects with subjective memory complaints resulted in a transient improvement in composite memory at week 4 in the group receiving the higher ET dose and time-dependent improvement in reaction time in both groups. A dose-dependent improvement in subjective prospective memory and sleep initiation was observed at a dose of 25 mg [72].

Oral administration of ET (5 mg daily) in the form of mushroom extract tablets to healthy participants and patients with MCI for up to 12 weeks improved their cognitive function. Subjects scoring 23–30 on the Mini-Mental State Examination, with an average age of 45, were supplemented with mushroom extract tablets containing 5 mg ET daily for 12 weeks. Individuals scoring less than or equal to 27 were suspected MCI patients. The supplemented subjects had higher blood ET levels than the placebo group; their verbal memory was significantly enhanced in comparison with the state before supplementation, while no improvement was seen in the placebo group. The improvement in cognitive function due to the supplementation was more significant in older individuals and those with severe cognitive impairment and lower blood ET concentrations [169]. It should be repeated, however, that mushrooms also contain other bioactive compounds, apart from ET, so the beneficial effect of mushroom consumption cannot be unequivocally ascribed to ET.

It is not obvious, however, what is the cause and what is the effect; that is, whether lower ET levels cause cognitive decline or are a consequence of neurodegeneration. Results of longitudinal studies speak in favor of the first possibility. A 5-year longitudinal study of cognitively normal elderly individuals revealed that low blood ET levels preceded cognitive decline and were associated with an accelerated rate of cognitive decline concerning attention, memory, executive function, visuomotor speed, and language [117]. Another longitudinal study in 1344 dementia-free elderly Japanese subjects revealed that lower ET levels predicted a much greater probability of developing all-cause dementia [70].

High blood levels of trimethylamine-N-oxide (TMAO) correlate with augmented risk of neurodegenerative diseases. TMAO is formed mainly by oxidation of gut-microbiota-derived trimethylamine (TMA) in the liver. A question has been posed whether microbial degradation of ET may contribute to the TMAO pool. However, bioinformatic analyses demonstrated that ergothionase, the enzyme responsible for ET degradation to TMA, is restricted to a small number of bacterial genera and is much less abundant than choline trimethylamine lyase, an enzyme that decomposes TMA. In humans, while ET supplementation (25 mg/day for 7 days) significantly increased plasma ET concentration, TMAO’s concentration was not elevated. These data obviate a significant contribution of ET consumption to TMAO levels in the body [170].

ET has been listed as a putative “anti-aging vitamin” [171] and has been approved as a supplement by regulatory authorities in the USA (GRAS) and in the European Union [17,35]. ET is believed by some researchers to be “a brain-penetrant agent safe for human consumption, that not only slows neurodegeneration but also promotes the genesis of new neurons and supports the functions of new and existing neurons” that “would be of immense value in the slowing and prevention of age-related diseases of the nervous system, especially for dementia” [21]. It has been emphasized that no adverse effects of ET administration were noted, even in a trial lasting for 56 weeks [73].

Are these apparently convincing data sufficient to broadly recommend ET supplementation for a general, especially elderly, population? In our opinion, such a recommendation should be preceded by further longitudinal randomized large-scale studies, like ATBC [172] and CARET [173] studies for β-carotene, recruiting patients in various age, ethnic, and nutritional profile groups.

## 8. Mechanisms of Ergothioneine Action

ET is a potent antioxidant, so the first emerging hypothesis links its protective effects against neurodegeneration to the antioxidant activity, as oxidative stress accompanies neurodegenerative diseases [12,13,14]. However, it has been argued that other mechanisms, both dependent on or independent of the antioxidant activity of ET, may also contribute to ET action.

### 8.1. Attenuation of Oxidative Stress

Interesting data concern mice haplodeficient in manganese-containing superoxide dismutase (SOD2), generated on the basis of the murine (Thy-1)-h[A30P]-α-synuclein transgenic line. These mice, having only half the normal SOD-2 activity, exhibited a significantly more advanced stage of synucleinopathy. This result shows that a weakening of the free radical scavenging capacity can augment the progression of PD [174]. Aβ may promote oxidative stress by formation of complexes with redox-active metals, such as copper. Such complexes are known to catalyze generation of superoxide and hydrogen peroxide [175]. Aβ1-42 oligomers are known to promote lipid peroxidation, as evidenced by increased levels of lipid peroxidation end products, 4-hydroxy-2-trans-nonenal and MDA, which are cytotoxic and may lead to neuronal death [176,177].

Attenuation of oxidative stress by ET has been demonstrated not only in vitro (especially when ET concentrations far exceeding those attainable in vivo are often used) but also in many in vivo experiments (Section 7.2). One may wonder how ET, present in micromolar amounts that are quite low in comparison with the main endogenous antioxidants, especially GSH and ascorbate in some cells, can significantly contribute to the antioxidant defense at the whole-body level. However, it can be an important antioxidant, taking into account its peculiar antioxidant behavior (Section 2) and the possibility of selective accumulation in some cells and subcellular compartments conditioned by OCTN1 distribution. ET can also act indirectly, enhancing the antioxidant level via activation of the Keap-nuclear factor erythroid 2-related factor 2 (Nrf2) pathway (Section 8.6).

### 8.2. Persulfidation

It has been suggested that the release of hydrogen sulfide H_2_S from ET is a predominant mechanism of its cytoprotective action, but the mechanism of this effect is not clear. ET increased the performance of mice; this effect was not observed in *Mpst^-/-^* mice. ET supplementation alone in myocyte cell culture media boosted mitochondrial respiration. Proteomic studies in mice identified 3-mercaptopyruvate sulfurtransferase (MPST), one of three enzymes known to produce H_2_S in mammals, as a direct molecular target of ET. MPST acts on 3-mercaptopyruvate to produce H_2_S and pyruvate in the mitochondria; thus, it may significantly contribute to electron flow and respiration. Proteome-wide thermal shift (a method dependent on ligand binding that affects the thermostability of the binding protein(s) within a proteome) identified MPST as a protein target of ET. In vitro assays demonstrated that ET bound to recombinant MPST with a dissociation constant (K_D_) of 3.2 mM. It has thus been proposed that ET binds to and activates MPST, and serves as a transient acceptor of H_2_S released by the enzyme [178].

Other authors have postulated that ET may be an alternative substrate for cystathionine gamma-lyase (CSE), producing H_2_S directly from ET. This reaction increases the persulfidation (the addition of sulfur onto a reactive thiol group of a cysteine residues, forming hydropersulfides—SSH) of more than 300 protein targets. Persulfidation was postulated to play an important regulatory role in AD and PD progression by interacting with such protein modifications as phosphorylation, *S*-nitrosylation, and succinylation; regulating key pathogenic proteins including tau, Aβ, and parkin; participating in the control of energy metabolism; protecting against oxidative stress; and attenuating inflammatory responses. These effects can affect the functional homeostasis and survival of neurons [160]. In mouse embryonic fibroblasts, thermal proteome profiling identified 14 potential binding targets for ET. Among these proteins, CSE was the only target that could potentially explain the effects of persulfidation. ET treatment increased mitochondrial respiration, ATP production, and cell growth while elevating the level of NAD^+^. An increase in the NAD^+^ level was observed both in vitro (in mouse embryonic fibroblasts) and in vivo (in *C. elegans* and rat muscle), and mainly seemed to be due to the activation of cytosolic glycerol-3-phosphate dehydrogenase by persulfidation [179].

It has been demonstrated that persulfidation and a concomitant increase in irreversible cysteine hyperoxidation decrease with aging, which enhances protein dysfunction and aggregation and may contribute to neurodegeneration [180]. Increased persulfidation may protect proteins from oxidative damage and contribute to the ET-evoked lifespan extension in *C. elegans* [179]. Although beneficial neuroprotective effects of persulfidation induced by low doses of hydrogen sulfide have been demonstrated [180,181], there is still no evidence for such effects in humans or in AD and PD models [182].

### 8.3. Inhibition of Protein Aggregation

In AD, the first pathological sign preceding tau hyperphosphorylation, gliosis and neurodegeneration is the accumulation of β-amyloid plaques (Aβ42) in the cortex [183]. It has been proposed that phosphorylated tau promotes accumulation of toxic Aβ and microtubule destabilization and drives the progression of the disease. Thus, decreasing the level of phosphorylated tau may alleviate the deleterious effects of Aβ [184]. Similarly, the accumulation of α-synuclein aggregates in Lewy bodies in the substantia nigra pars compacta region of the midbrain is the histopathological hallmark of PD [185].

ET was demonstrated to dose-dependently inhibit α-syn aggregation and fibrillogenesis in vitro, 250 μM ET causing almost complete inhibition. ET not only prolonged the lag phase of α-syn fiber formation, but also disrupted mature, about 9 nm long fibers into rod-shaped, about 4 nm long aggregates, which were less toxic. Direct interaction of ET with α-syn pentamer through van der Waals and electrostatic forces was demonstrated by molecular dynamics simulation. Such interactions can disrupt the structural stability of the preformed α-syn pentamer [144].

ET reduced protein aggregation in both PD and AD models, including Aβ, tau, and α-syn [112]. It raises the intriguing possibility that ET may also be protective against other disorders driven by protein misfolding, such as HD (Huntingtin aggregation) and ALS (TDP-43 aggregation) [19] or even prion diseases. This question has not yet been explored.

Activation of glycogen synthase kinase-3β (GSK-3β) enhances the expression of Aβ precursor protein (APP), BACE1, and nicastrin that are involved in the generation of Aβ. Activation of GSK-3β and increased APP, BACE1, and γ-secretase expression were observed in cultured hippocampal neurons exposed to Aβ25–35 oligomers. ET inactivated GSK-3β and suppressed Aβ toxicity, reducing the expression of *p*-tau and proteins involved in Aβ production, and ameliorating neurotoxicity. Supposedly, ET activates the PI3K-Akt pathway and thus augments the phosphorylation of *p*-GSK-3β at serine 9 [143].

Apart from preventing aggregation, ET can stimulate Aβ clearance. ET administration to 5× FAD mice (50 mg/kg 3 times per week for 8 weeks) significantly decreased the number of large Aβ deposits or plaques in the neuroretina and elevated the count of IBA1(+)ve blood-derived phagocytic macrophages. These observations suggest that ET stimulates the clearance of Aβ by IBA1(+)ve macrophages derived from blood or via perivascular drainage [154].

### 8.4. Prevention of Apoptosis

Aβ and α-syn aggregates can drive oxidative stress and mitochondrial dysfunction [19], which may activate the intrinsic apoptotic pathway, a mitochondria-dependent process orchestrated by proteins of the Bcl-2 family. In the MPTP mouse model of PD, ET decreased the rate of apoptosis, increasing the expression of Bcl-2, lowering the expression of Bax and caspase-3, and decreasing the level of cleaved caspase-3. ET treatment also attenuated the MPTP-induced downregulation of VMAT2, DDC, TH, and DJ-1-nuclear receptor-related factor 1 (Nurr1) proteins. Nurr1 is an essential pro-survival factor for dopaminergic neurons [152].

In Aβ-treated SH-SY5Y cells and cultured primary hippocampal neurons, overexpression of GSK3β can trigger apoptosis. ET was found to inhibit the Aβ25–35-induced activation of GSK3β and attenuate the decrease in cell viability [143].

On the other hand, ET was suggested to eliminate senescent neuronal cells, thus exhibiting a senolytic action [83,186,187].

### 8.5. Reduction in Neuroinflammation

Neuroinflammation, consisting in immune activation of astrocytes and microglia, is an important pathogenic factor for both PD and AD [188,189,190,191]. ET, apart from ameliorating oxidative stress (a consequence of inflammation), was reported to exert a direct anti-inflammatory effect. In PD, α-syn aggregation enhances the reactivity of astrocytes, resulting in augmented release of pro-inflammatory cytokines [191]. Overactivation of astrocytes and microglia can activate the Nod-like receptor protein 3 (NLRP3) inflammasome, which leads to the death of neurons [192]. In AD, activated astrocytes release huge amounts of pro-inflammatory cytokines, such as IL-1, IL-6, and IL-12, which have neurotoxic actions [193,194]. In the further stages of AD, the overactivation of microglia contributes to the dysfunction and loss of synapses [195].

The neuroprotective effects of ET may involve modulation of neuroinflammation [19]. ET inhibited the release of pro-inflammatory cytokines by cultured microglia [100]. Alleviation of neuroinflammation may underlie ET’s antidepressant effects [166]. Exposure of human BBB endothelial cells hCMEC/D3 to 7-ketocholesterol increased RNA expressions of IL-1β, IL-6, and IL-8. This effect was significantly attenuated by ET [147].

Computational analyses showed that ET targets molecules, such as tumor necrosis factor (TNF), AKT Serine/Threonine Kinase 1 (AKT1), caspase 3 (CASP3), and Interleukin 6 (IL-6), which points to the possibility of regulation of some inflammation-related signaling cascades. ET considerably reduced the production of pro-inflammatory cytokines by BV2 microglia cells stimulated with lipopolysaccharide, modulating the phosphoinositide 3-kinase/protein kinase B (AKT) and nuclear factor kappa-light-chain-enhancer of activated B cell (NF-κB) signaling pathways [196].

Administration of ET-rich *Hericium erinaceus* to APP/PS1 mice caused a decrease in the levels of pro-inflammatory cytokines (IL-1β, IL-6, and TNF-α) in blood serum and also in the brain [160]. ET was thus proposed to be a functional food ingredient for managing neuroinflammation [196].

### 8.6. Effects of Ergothioneine on Signaling Pathways

Apart from inhibiting pro-inflammatory (Section 8.5) and proapoptotic (Section 8.4) pathways, ET was also demonstrated to affect other cellular signaling pathways. Attenuation of oxidative stress by ET may affect some of these pathways. Oxidative stress inactivates the protein tyrosine phosphatase 1B (PTP1B). PTP1B inhibits Nrf2 activity by promoting its tyrosine phosphorylation, its exclusion from the nucleus, and its subsequent degradation [197]. ET blocked the olanzapine-induced upregulation of PTP1B expression in brain tissues, thus preserving Nrf2 activity and abolishing the olanzapine-induced synaptic and cognitive deficits [85]. Oxidative stress via *S*-nitrosylation can also inhibit the function of parkin. This protein is a key regulator of mitophagy; therefore, its inactivation impairs the effective clearance of dysfunctional mitochondria [198]. ET can prevent this effect by attenuating oxidative stress.

In HaCaT keratinocytes, etoposide treatment decreased the levels of phosphorylated phosphoinositide 3-kinase (*p*-PI3K) and phosphorylated protein kinase B (*p*-Akt), whereas ET upregulated these levels [199]. In differentiated SH-SY5Y cells and primary cultured neurons, ET ameliorated Aβ toxicity and inhibited tau protein phosphorylation at serine 396 evoked by Aβ, probably via preventing GSK-3β activation. Activation of the PI3K-Akt pathway enhances phosphorylation of GSK-3β at serine 9. It has been postulated that ET increases the phosphorylation of GSK-3β at serine 9 by activating the PI3K-Akt pathway. However, the mechanism of this activation is unclear and requires further study [143]. Docking and dynamics simulations have revealed the strong and stable binding of ET to several proteins involved in inflammatory signaling, including Akt, which may be a premise for its postulated application as a functional food ingredient inhibiting neuroinflammation [196,200]. The neuroprotective potential of ET in experimental ischemic stroke (reduction in infarct volume, improvement of cerebral perfusion and attenuation of glial cell activation) was also found to be mediated by the PI3K/Akt/Nrf2 pathway [201].

Several studies reported activation of Nrf2, the “master regulator” of endogenous antioxidant defense, by ET. Nrf2 activation by ET has been reported in various cell types [199,202,203,204,205,206,207,208]. The Nrf2-Keap1 signaling pathway is important in protection against neurodegenerative diseases; its promotion of antioxidant enzyme gene expression can effectively reduce ROS-induced dopaminergic neuron damage. ET was found to be a key regulator of DJ-1, a protein DJ-1 promoting the detachment of Nrf2 from its negative regulator, Keap1, thereby enabling its nucleation. ET was reported to restore DA levels by activating the Nurr1 axis [152]. Moreover, it has been shown that DJ-1 enhanced the transcriptional activity of Nurr1 to promote dopamine synthesis and transport. Therefore, activation of DJ-1 can constitute a potential target in PD therapy [209]. In mouse midbrain dopaminergic neuron N9D cells, ET downregulated Keap1 and increased the levels of Nrf2 [152], so the upregulation of the levels of endogenous antioxidants seems to be the main mechanism of the Nrf2-mediated protective action of ET in neurons.

The enhanced antioxidant effect through the combination of ET and lactoferrin in the protection of mouse neuroblastoma N2a cells against Aβ25–35-induced cytotoxicity has been ascribed to Keap1/Nrf2-mediated mechanisms [164]. In retinal pigment epithelial cells, ET protection against oxidative stress was dependent on the activation of the Nrf2 pathway [202].

In turn, the inhibition of osteoarthritis by ET involved hindering NF-κB activity by activating the SIRT6 pathway [210].

In a human coronary artery endothelial cell (HCAEC) hypoxia model, ET reduced the expression of sFlt-1, GLRX, and Wnt5a, suggesting that ET protects the heart from infarction-inflicted damage by reducing protein *S*-glutathionylation via the NF-κB-dependent Wnt5a-sFlt-1 pathway [211]. A similar mechanism could operate in other cell types.

It has been suggested that ET promotes neurogenesis and neuronal differentiation and enhances the effects of neurotrophic factors [143,195], which may also have importance in the protection against neurodegeneration and its therapy.

Cognitive function and hippocampal neurogenesis were better in mice fed a control diet than in animals fed an ET-free diet. ET supplementation, which allows the ET level typical for a controlled diet to be achieved, thereby reversing these effects. The ET-evoked recovery was blocked by inhibition of the neurotrophic factor receptor tropomyosin receptor kinase B (TrkB), which suggests that TrkB is involved in the action of ET. Phosphorylated TrkB was also found in extracellular vesicles isolated from human blood serum. The extent of TrkB phosphorylation was greater in volunteers given ET than in control subjects and showed positive correlation with ET concentration in the serum and several scores evaluating cognition [167].

In Neuro2a cells, a reduction in ET uptake by knockdown of OCTN1 upregulated the oxidative stress marker heme oxygenase 1 and Sox2 while downregulating the neurite outgrowth marker GAP43 [98]. ET inhibited the proliferation of neuronal stem cells but promoted their differentiation. The latter effect involved modulation of expression of basic helix-loop-helix transcription factors; its mechanism was not revealed but was independent of the antioxidant action of ET [99]. Microglial cells from octn1^−/−^ mice decreased cellular hypertrophy upon stimulation with LPS [100]. Treatment with ET upregulated neurotrophin-3, neurotrophin-5, and the phosphorylated mammalian target of rapamycin (mTOR) in hippocampal neurons [148]. ET also enhanced the expression of neurotrophin 4/5 in cultured neural stem cells and in the mouse hippocampus [166].

ET was also found to activate the mTOR signaling pathway in cultured neural stem cells and the mouse hippocampus [210]. It has been reported that the activation of the mTOR signaling pathway is involved in the action of rapid antidepressants [212].

Apparently, this direct and indirect interference with signaling pathways may be the main mode of the neuroprotective actions of ET.

### 8.7. Modification of Gut Microbiota

Brain function is modulated by gut microbiota that produce bioactive metabolites, including short-chain fatty acids and antioxidants, which exert cytoprotective effects. These compounds can cross the BBB and enhance synaptogenesis, promote neurite outgrowth, and improve memory [213,214]. Among these, it has been documented that short-chain fatty acids attenuate neuroinflammation, help maintain blood–brain barrier integrity, and inhibit amyloid protein aggregation [215]. Human studies have revealed decreased levels of *Eubacterium rectale*, *Bacteroides fragilis*, *B. hungatei*, *B. proteoclasticus*, and *Lactobacillales* and increased levels of *Helicobacter pylori*, *Escherichia*/*Shigella* ratios, *K. pneumoniae*, *B. fragilis*, *E. lenta*, and *Oscillo192C-spirales* in patients with AD [216,217,218,219]. In PD, decreased levels of *Prevotella*, *Lactococcus* spp., *Blautia*, *Roseburia*, *Fecalibacterium*, and *Prevotellaceae* and increased levels of *Verrucomicrobia*, *Mucispirillum*, *Porphyromonas*, *Lactobacillus*, *Parabacteroides*, *Ralstonia*, and *Enterobacteriaceae* were reported [220,221,222,223,224]. In amyotrophic lateral sclerosis, the lowered abundance of *Firmicutes*, *Prevotella* spp., *Eubacterium rectale*, and *Roseburia intestinalis* and an elevated abundance of *Bacteroidetes* were revealed [225,226,227]. In Huntington’s disease, decreased levels of *Bilophila* and *Firmicutes* and increased levels of *Intestinimonas* and *Bacteroidetes* were reported [156,228]; these may either contribute to the development of neurodegenerative diseases or be their secondary effects.

However, probiotics administered in animal models of neurodegenerative diseases have shown a range of positive effects, including inhibiting dopaminergic neuron death, decreasing brain inflammation, reducing Aβ plaque formation, and increasing cognitive function [210]. Studies of transplantation of fecal matter further suggest that the gut microbiome may play a role in the pathogenesis of PD [19]. Fecal matter transplants from Thy1-ASyn (alpha-synuclein-overexpressing) mice to germ-free mice promoted motor deficits and neuroinflammation [229]. Fecal matter transplants from PD patients to A53T PD mice (a mouse PD model) enhanced inflammation, DA neuronal loss, and abnormal αSyn deposits [230]. In contrast, fecal matter transplants from healthy humans ameliorated neurodegenerative changes in the MPTP-induced mouse model of PD [231].

Treatment of APP/PS1 mice with ET (40 mg/kg) improved cognitive function, reduced neuronal damage, lowered Aβ accumulation, attenuated microglial overactivation, decreased the level of TNF-α, and partially normalized the homeostasis of neurotransmitters and chemokines in the brain. This treatment altered the composition of the gut microbiome in APP/PS1 mice, increasing the amounts of Alistipes, Clostridiales_unclassified, and Peptococcaceae_unclassified while enhancing the formation of butyric acid, isobutyric acid, and valeric acid [156]. In the same mouse model of AD, the administration of pure ET (1.44 mg/kg) and ET-rich aqueous extracts from the mushroom *Hericium erinaceus*, containing either 1.44 mg ET/kg or 5.76 mg ET/kg, enhanced cognitive function, improved spatial learning and memory, and inhibited Aβ aggregation and tau phosphorylation. Administration of a low dose of the extract lowered the abundance of Fusobacteriota and Proteobacteria while elevating the abundance of *Lactobacillus*. ET supplementation significantly augmented the abundance of Alloprevotella, and the high-dose extract increased the abundance of *Dubosiella*. Treatment with either the extract or pure ET decreased the abundance of *Muribaculum*. Low-dose extracts and pure ET increased the abundance of *Lactobacillus*. These data suggest that ET and the *H. erinaceus* extract may exert neuroprotective effects at least partly via modulation of the gut microbiome, including an increase in the abundance of *Lactobacillus* [232]. It has been demonstrated that *Lactobacillus* can enhance acetylcholine levels; this effect may play a role in the improvement of cognitive function and amelioration of AD-related pathology [233,234]. However, this suggestion remains speculative, as the high dose of the extract exerted a neuroprotective effect without a significant effect on the abundance of *Lactobacillus*, which may indicate that these effects may be parallel but independent.

Although these data point to the beneficial effects of ET on the gut–brain axis in AD, they do not prove that the brain effects of ET were mediated by gut microbiota. Demonstration of the mediating role of gut microbiome in the neuroprotective action of ET would require further studies and more precise experiments. Moreover, interspecies differences in the composition of the gut microbiome suggest care should be taken in the interpretation of data obtained from animal models.

## 9. Conclusions and Perspectives

Cellular and animal experiments, as well as human epidemiological and interventional data, strongly suggest that ET is an interesting natural neuroprotective agent for preventing and slowing PD progression. ET is thus a promising candidate for prophylaxis and therapy of neurodegenerative diseases, especially since it seems safe for human consumption, and its chemical stability facilitates its storage and delivery. The published data suggest that ET supplementation can be broadly recommended for the general population, especially older adults, to prevent, delay, and limit neurodegeneration. ET can be taken as a dietary supplement or as a component of functional foods. The consumption of mushrooms, both fresh and processed (especially dried), can be a reasonable choice.

Still, the mechanisms of ET action are not sufficiently understood and require elucidation. The data on the beneficial effects of ET require substantiation. Small-scale studies should be followed by large-scale and long-term randomized trials using pure ET to eliminate the effects of other compounds present in food products. The database of ET contents in food products should be enlarged, e.g., through a comparison of a broad range of edible mushrooms. Optimal conditions for the cultivation of mushrooms should be chosen to provide the highest ET contents.

## Figures and Tables

**Table 1:** Ergothioneine (ET) levels in human blood, basal and after supplementation.

Population	No. of Subjects, Characteristics	Material Studied	Basal ET Level	ET Supplementation	ET Level After Supplementation	Reference
Saudi Arabia	Males 1–10 y, *n* = 150 11–18 y, *n* = 100 19–51+, *n* = 150	Blood cell sediment	65–87 μM 157–161 μM 100–131 μM			[65]
Singapore	*n* = 5	Blood plasma Erythrocytes	0.468 ± 0.031 μM 5.60 ± 5.94 μM			[66]
Singapore	<65 y 65–70 y 71–75 >75 y, *n* = 50	Blood plasma	0.443 ± 0.01 μM 0.414 ± 0.030 μM 0.393 ± 0.031 μM 0.276 ± 0.021 μM			[67]
Singapore	21–35 y, *n* = 15	Blood plasma Whole blood Urine	ca 1.03 μM 99.3 ± 74.0 μM, ca 13.3 nmol/mol creatinine	25 mg/d, 7 d	ca 2.93 μM 94.4 ± 72.0 μM, ca 37.6 nmol/mol creatinine	[63]
NSW, Australia	55–85 y, *n* = 444 *n* = 215 Females *n* = 229 Males	Blood serum	1.02 μM (0.79–1.35 μM) 0.99 μM (0.75–1.35 μM) 1.04 μM (0.80–1.38 μM)			[68]
Japan	30–59 y, *n* = 14	Blood plasma	3.42 ± 1.74 μM	5 mg/d, 56 d 10 mg/d, 56 d	5.52 ± 1.97 μM 7.99 ± 4.53 μM	[69]
Japan	30–59 y, *n* = 28	Erythrocytes	594 μM			[69]
Japan	40–64 y, *n* = 50	Blood plasma	4.07 μM	8 mg/d, 56 d	9.38 μM	[69]
Hisayama, Japan	65–101 y, *n* = 1344	Blood plasma	0.693 μM			[70]
Nunavik, Québec	Inuit, 16–60+ y, *n* = 1291 Females Males	Whole blood	403.4 μM 457.9 μM 358.0 μM			[59]
Québec, Nunavik	Non-indigenous laboratory volunteers, 21–56 y, *n* = 10	Whole blood	98.6 μM			[59]
Japan	Sleep complaints, 40–75 y, *n* = 50	Blood plasma	2.97 μM	20 mg/d, 4 wk	9.51 μM	[71]
Australia	Self-reported memory complaints, 55–79 y, *n* = 47 *n* = 49	Blood plasma	1.19 μM 1.09 μM	10 mg/d, 16 wk 25 mg/d, 16 wk	ca 6.2 μM ca 17.7 μM	[72]
Singapore	60–90 y, mild cognitive impairment, *n* = 14	Blood plasma	0.86 μM	25 mg, 3 times per week 28 wk 56 wk	3.96 μM 6.86 μM	[73]
Australia	No cognitive impairment, 65–90 y, *n* = 65 Aβ− *n* = 35 Aβ+	Blood serum	0.955 ± 0.871 μM 0.958 ± 0.610 μM			[74]
Singapore	Neovascular age-related macular degeneration, *n* = 100	Blood serum	1.169 ± 0.081 μM			[75]

Aβ−, low neocortical amyloid load; Aβ+, high neocortical amyloid load.

## Data Availability

No new data were created or analyzed in this study. Data sharing is not applicable to this article.

## Abbreviations

The following abbreviations are used in this manuscript:

6-OHDA	6-Hydroxydopamine
Aβ	Amyloid β peptide
AD	Alzheimer’s disease
ALS	Amyotrophic lateral sclerosis
APP	Aβ precursor protein
BBB	Blood–brain barrier
bw	Body weight
DA	Dopamine
ET	Ergothioneine
GSH	Glutathione
GSSG	Oxidized glutathione (glutathione disulfide)
hs-CRP	High-sensitivity C-reactive protein
IL	Interleukin
HD	Huntington’s disease
MCI	Mild cognitive impairment
MDA	Malondialdehyde
MPP^+^	1-Methyl-4-phenylpyridinium
MPTP	1-Methyl-4-phenyl-1,2,3,6-tetrahydropyridine
MPST	3-Mercaptopyruvate sulfurtransferase
mTOR	Mammalian target of rapamycin
OCTN1	Organic cation transporter novel 1
PD	Parkinson’s disease
PTP	Protein tyrosine phosphatase
ROS	Reactive oxygen species
SOD	Superoxide dismutase
α-syn	α-Synuclein
TMA	Trimethylamine
TMAO	Trimethylamine-N-oxide
TNF	Tumor necrosis factor
TrkB	Tropomyosin receptor kinase
